# Ring-Size Effects on the Stability and Spectral Shifts of Hydrogen Bonded Cyclic Ethers Complexes

**DOI:** 10.1038/s41598-017-18191-3

**Published:** 2018-01-24

**Authors:** Shanshan Tang, Narcisse T. Tsona, Lin Du

**Affiliations:** Environment Research Institute, Shandong University, Shanda South Road 27, 250100 Shandong, China

## Abstract

In order to investigate ring-size effects on the stability and spectral shifts of hydrogen bonded cyclic ethers complexes, the strength of hydrogen bonds in gas phase complexes formed between 2,2,2-trifluoroethanol (TFE) and selected cyclic ethers were examined using FTIR spectroscopy. TFE was chosen as hydrogen bond donor in these complexes, while trimethylene oxide (TMO), tetrahydrofuran (THF) and tetrahydropyran (THP) were selected as hydrogen bond acceptors. Comparable OH-stretching red shifts were observed in the three kinds of complexes. The difference of red shifts is so small (<7 cm^−1^) for TFE−TMO/THF/THP complexes that one can conclude that their stabilities and the strength of the hydrogen bonds are nearly similar and do not show any marked dependence with the ring size of the hydrogen bond acceptor. The equilibrium constants for the complexation were determined, and atoms-in-molecules (AIM) and natural bond orbital (NBO) analyses were performed to further investigate the intermolecular interactions. Regardless of the ring size, hydrogen bonds in the complexes showed similar strength, in agreement with the observed OH-stretching red shifts.

## Introduction

In the atmosphere, oxygenated volatile organic compounds (OVOCs) from both anthropogenic and biogenic sources undergo various chemical and physical processes, leading to their transformation or removal from the atmosphere. Ethers, as one of the main OVOCs, which are used as industrial solvents and additives to unleaded gasoline to increase the octane rating, are emitted to the atmosphere, where they contribute to the formation of photochemical smog. Ethers have lower vapor pressure and are more reactive in the atmosphere than alkanes of similar chain length generally^1,2^. Low-volatility multifunctional OVOCs condense onto existing atmospheric particles and hence increase the organic fraction content of secondary organic aerosols (SOA). The contribution of OVOC oxidation to SOA formation is of interest with respect to its potential impacts on air quality, human health, and climate^2,3^. Given the widespread use of oxygenated compounds as solvents and additives, it is important to characterize the sink of these compounds. Trimethylene oxide (TMO), tetrahydrofuran (THF) and tetrahydropyran (THP) are typical cyclic molecules that exhibit interesting features^4–6^. Paclitaxel (Taxol) is an example of a natural product containing a TMO ring. Taxol has become a major point of interest for researchers due to its unusual structure and success in the involvement of cancer treatment. The TMO ring is an important feature used for the binding of microtubules in structure activity^4^. THF is a promising green solvent that is relatively non-toxic and miscible with water over a wide range of reaction conditions. THF has been explored as a miscible co-solvent to aid in the liquefaction and delignification of plant lignocellulosic biomass for production of renewable platform chemicals and sugars as potential precursors to biofuels^5^. Furthermore, THF is a structural unit of the phosphate deoxyribose backbone of DNA^7^. THP is part of the chemical composition of antimicrobial compounds and metabolites^8^.

In recent years, a great interest in the chemistry of cyclic ethers has emerged. Also, cyclic ethers as O-electron donors in O–H∙∙∙O hydrogen bonding have been widely studied^9–11^. The exact structure of THF has been studied using electron momentum spectroscopy, triple differential cross section measurement, and high-resolution photo absorption spectroscopy^12–14^. THF is nonplanar and exhibits an internal motion called pseudo-rotation, which gives rise to several local minima in the potential energy surface. Many experimental and computational methods have been employed to explore the structure of THF and to determine the barrier height for the pseudo-rotation. Among all conformers, those having C_2_ and C_s_ symmetry are the most stable^9,15^. The intermolecular hydrogen bond interaction involving THF molecule has been studied due to its interesting geometry. For example, the formation of 1:1 complex of 2,2,2-trifluoroethanol (TFE) with THF has been observed in the gas phase, which results in a decrease in the intensity of the alcohol band centered at 3657 cm^−1^, the characteristic free OH stretching vibration, and the appearance of a new broader band around 3440 cm^−1^ due to a bonded OH stretching vibration, characteristic of the complex. As expected, adding more THF causes a further decrease in the free OH band and the rising of the bonded OH band^11^. The vibrational spectrum of the 1:1 aniline–tetrahydrofuran complex, formed in a pulsed supersonic jet, has been observed in the NH stretching region. The optimized geometry showed that one of the amino hydrogens of aniline interacts with the oxygen of tetrahydrofuran, and forms a complex with 37 kJ mol^−1^ binding energy^16^. In addition, the complexes of THF with donor molecules such as H_2_O, N-substituted caproamides, and phenol have been characterized^10,17–19^. However, the complexes with TMO and THP molecules have received much less attention than complexes with THF. Complexes of TMO with donor molecules HF, HCl, and those of THP with H_2_O and HCl have been characterized by employing computational methods^20–23^.

Formation of hydrogen bonded complexes using methanol (MeOH) and 4-fluorophenol as hydrogen bond donors and 39 ethers of widely different structures as hydrogen bond acceptors, including TMO, THF and THP was explored in CCl_4_ at 298 K by FTIR spectrometry^24^. The red shifts of the OH-stretching transition in MeOH–TMO, MeOH–THF and MeOH–THP complexes were in the range of 157–158 cm^−1^. For complexes with 4-fluorophenol as hydrogen-bond donor, the red shifts only differed by <6 cm^−1^ ^24^. Besides, this observation agrees with the results obtained when comparing the N–H∙∙∙O hydrogen bonds in the study of complexes of N-methylpropionamide (NMP) with THF and THP using three different spectroscopic techniques: fundamental IR, NIR and ^1^H NMR in CCl_4_ solutions. This difference in stability of THF and THP complexes with an amide can be attributed to the presence of an additional CH_2_ group in THP, i.e. to the more expressed steric hindrance in THP^25^. Furthermore, blue-shifting C-H∙∙∙O hydrogen bonded complexes between chloroform and three small cyclic ketones (cyclohexanone, cyclopentanone, and cyclobutanone) have been identified by use of FTIR spectroscopy in CCl_4_ solution at room temperature. The shifts of the C-H stretching fundamental of chloroform in the three complexes are +1, +2, and +5 cm^−1^, respectively. Spectral analysis reveals that the complex with cyclohexanone is the most stable, and the stability decreases with the ring size of the cyclic ketones^26^. Studies of interactions of 3,4-dinitrophenol (DNP) with cyclic ketones and MeOH with lactams identified that the strength of the hydrogen bond increases with the ring size^27,28^.

Cyclic ethers are used as the oxygen centered hydrogen bonding acceptors in the present study, because the OH-stretching fundamental frequencies of these molecules are different for four-, five-, and six-membered systems. To confirm the effect of ring size on hydrogen bond, we extend our investigation to complexes of TFE with cyclic ethers, TMO, THF and THP. Infrared (IR) spectroscopy is one of the most powerful methods available for studying both the structure and dynamics of such complexes. In particular, the frequency of the OH stretching mode is very sensitive to the type and strength of hydrogen bonding present. Special emphasis is put on hydrogen bond-induced vibrational OH-stretching shifts. Such shifts are typically bathochromic and we use the colloquial term “red shift”, which would be more appropriate in the visible range. Formation of a hydrogen bond generally causes red shift in the OH stretching band compared to the band of free OH. Fluctuations in hydrogen bond strength lead to distribution of the OH stretching frequency, which results in broadening the OH absorption band. The gas-phase IR spectra of the TFE–TMO/THF/THP complexes were measured at room temperature to ensure that the complexes produced were thermally stable. The equilibrium constant upon complexation was determined by combining the experimental integrated absorbance and the computational IR intensity of the OH-stretching transition band of the complex. The atoms-in-molecules (AIM) and Natural Bond Orbital (NBO) analyses were used to determine the electronic densities and hydrogen bond interactions, and to explain the donor-acceptor charge delocalization between the lone pair of the acceptor and the antibonding orbital of the donor in the studied complexes, respectively.

## Results and Discussion

### Description of the monomers

Geometry optimization of the monomers and complexes was performed at the B3LYP and B3LYP-D3 levels of theory using the aug-cc-pVTZ basis set. Although two stable conformers, the *trans*-conformer and *gauche*-conformer, were found for TFE, only the *gauche*-conformer was observed the gas-phase IR spectrum^29^. Henceforth, only the TFE *gauche*-conformer will be considered in the remainder of this study. THF, the hydrogen bond acceptor, possesses a global minimum C_s_ symmetry (envelope) and a local minimum C_2_ symmetry (twisted). The observed results shown a dominance of the local minimum energy structures under the experimental conditions^12^. It has been demonstrated that C_2_ conformer is more stable than C_s_ conformer in previous studies^9,15^. In the present study, complexes with both conformers were optimized and it was found that the C_s_ conformer gets transformed into the C_2_ conformer during optimization, which is in agreement with the p-cresol−THF complex^9^. Hereby, in all cases only the C_2_ conformers of THF were taken for further calculations. The structure of the target allows for varying degrees of interaction between the lone-electron pair and the carbon ring structure^13^. The relatively flat THF molecule becomes the chair conformation of THP because of the increase of the interaction between the non-bonding lone-electron pair and the carbon frame. It has been well established that THP exists in its lowest energy C_s_ symmetry chair conformation (THP-c) in gas phase^13^. The boat conformations of THP (THP-b1 and THP-b2) have been optimized, as shown in Figure S1 in the supplementary information. The energies of THP-b1 and THP-b2 are all much higher than the energy of THP-c (>24 kJ mol^−1^), and hereby, the complexes with boat conformation of THP are not considered in the present study (see Table S1 in the Supplementary information).

### Optimized geometries and formation energies of the hydrogen bonded complexes

**Figure 1:**
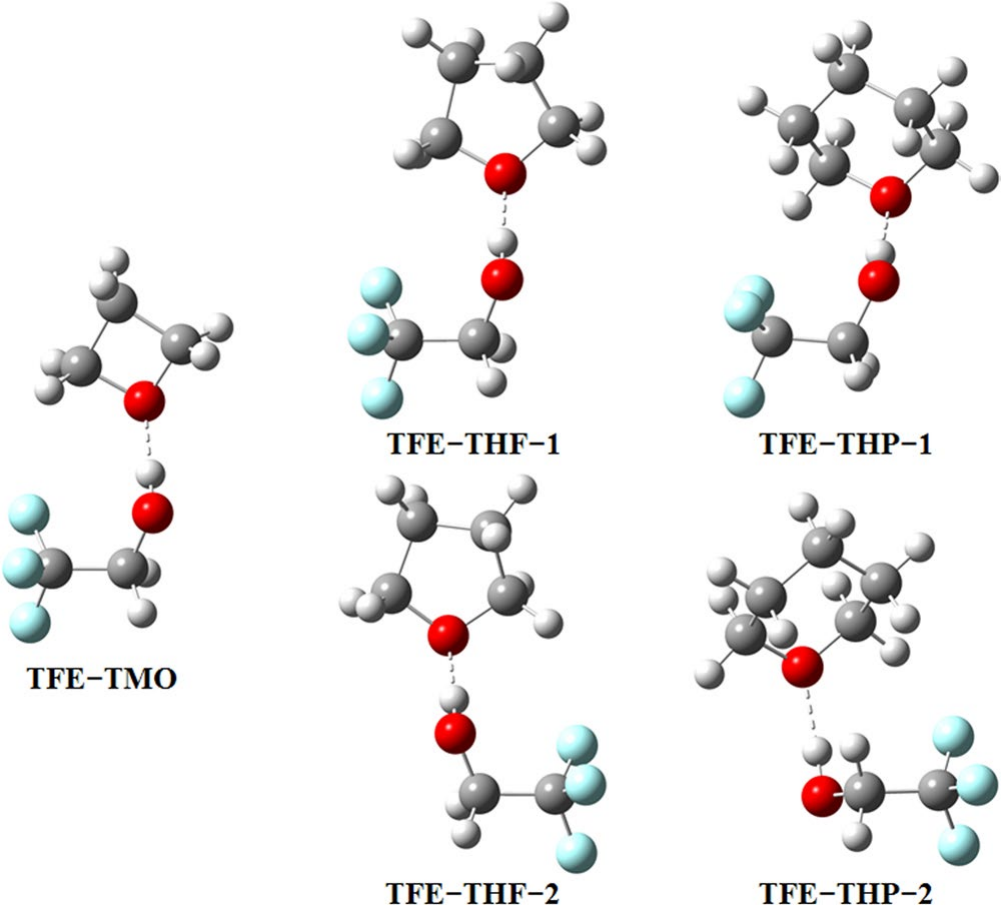
Structures of the most stable complexes optimized using the B3LYP-D3/aug-cc-pVTZ method.

In the investigated complexes, there is no doubt that the TMO has one conformation with TFE, since the four-membered ring shows a planar conformation^20^. In the case of asymmetric THF and THP, two stable structures were obtained respectively, where the TFE showed different orientations with respect to THF/THP, namely TFE–THF-1, TFE–THF-2, TFE–THP-1 and TFE–THP-2. Fully B3LYP-D3/aug-cc-pVTZ optimized structures of these complexes in their most stable conformations are reported in Fig. 1, while selected geometric parameters are shown in Tables 1 and S2. There is a sizable elongation of the OH bond due to the complex formation between the OH group of TFE and the oxygen of the cyclic ether. Accordingly, the OH bond length upon complex formation increases by 0.0169 Å for TFE–TMO, slightly larger than the change in other complexes. These elongations reflect the hydrogen bond strength which is stronger in the TFE–TMO complex than in other complexes, in agreement with literature results^30,31^. It may be attributed to the electronic density transfer from the proton acceptor to the proton donor due to the dominant stabilizing role of the dispersion forces^28^. The importance of steric effects on the hydrogen bond basicity of ethers is well established and explains that the more crowded the ether is, the less basic the alkyl ethers^32^. There are two opposite effects depending on the basicity of ethers. On one hand, the electronic effect of the alkyl group (methylene in this study) increases the hydrogen bond formation enthalpy and basicity. On the other hand, the steric effects may increase the hydrogen bond formation entropy and reduce the basicity. Considering the two effects, the non-monotonic variation of hydrogen bond basicity of cyclic ethers varies with the ring size in the following order: four- > five- > six- > three-membered rings^24^. Compared with our previous study about the TFE–EO complex, it can be noticed that the change in the OH bond length for TFE–EO is much smaller than that of TFE–TMO, which somewhat results from the instability of the three-membered ring based on the basicity order given by cyclization: oxetane > tetrahydrofuran > tetrahydropyran > oxirane^24^. In addition, it is shown that, as the intermolecular hydrogen bond angle approaches 180°, the charge transfer energy increases^33^. The analysis of Table 1 shows that the angles deviate within 5° and 9° from the ideal linear orientation for TFE–THF and TFE–THP, which is similar to the deviation observed in TFE–TMO. The three-membered ring has a larger deviated angle than other rings, which agrees very well with the change in the OH bond length. This indicates that similar hydrogen bond strengths are obtained in the four-, five- and six-membered rings complexes, and they are more stable than the three-membered ring complex. This can be seen from the discrepancy of basicity in a previous investigation^24^.

**Table 1:** Optimized geometric parameters of the TFE−cyclic ether complexes, calculated at the B3LYP-D3/aug-cc-pVTZ level of theory. Angles are in degrees and bond lengths are in Å.

Conformer	*r*_(OH)_^a^	Δ*r*_(OH)_^b^	*r*_(HB)_^c^	*θ*_(HB)_^d^
TFE–EO^e^	0.9761	0.0131	1.8027	168.7
TFE–TMO	0.9800	0.0169	1.7480	175.7
TFE–THF-1	0.9798	0.0167	1.7445	176.5
TFE–THF-2	0.9796	0.0166	1.7465	175.0
TFE–THP-1	0.9792	0.0161	1.7721	170.8
TFE–THP-2	0.9774	0.0144	1.7678	171.2

^*a*^OH bond length. ^*b*^Δ*r*_(OH)_ = *r*_complex_ − *r*_TFE_, is the change in the OH bond length upon complexation. ^*c*^Intermolecular hydrogen bond distance. ^*d*^Intermolecular hydrogen bond angle, i.e., *θ*_(O−H∙∙∙O)_.^*e*^ ref.^34^.

Tables 2 and S3 gather the computed energies pertaining to the formation of the different structures of the complexes, as well as the equilibrium constants at 298 K. Table 2 shows that the TFE–EO complex is the least stable among the studied complexes giving its least negative energies. Little differences were obtained in binding energies (difference <1.9 kJ mol^–1^) of the TFE–TMO/THF/THP complexes, similar to the difference between the O–H∙∙∙O and O–H∙∙∙S hydrogen bonded complexes (differences <2.3 kJ mol^−1^)^34^. Our calculations show that TFE–THF-1 is 0.2 kJ mol^−1^ more stable than TFE–THF-2, while TFE–THP-1 is 1.1 kJ mol^−1^ more stable than TFE–THP-2. Both the TFE–THF-1 and TFE–THP-1 conformers have very similar configurations with TFE–TMO. Although there were small differences in the TFE–TMO/THF/THP energies, similar hydrogen bond strengths were observed for these complexes. This is in agreement with the observation from a previous study on the structure and relative stability of methanol complexes with various cyclic ketones with three- to seven-membered rings^28^. Therefore, it is remarkable that the size of the ring has little effect on hydrogen bonding.

**Table 2:** Binding energy (*BE*), enthalpy of formation (${\rm{\Delta }}{H}_{calc}^{\theta }$ at 298 K), Gibbs free energy of formation (${\rm{\Delta }}{G}_{calc}^{\theta }$ at 298 K) and equilibrium constant (${K}_{eq}^{{\rm{calc}}}$ at 298 K) for the TFE−EO/TMO/THF/THP complexes. Calculations were performed with the B3LYP-D3/aug-cc-pVTZ method^a^.

Conformer	*BE* ^b^	ZPVE	BSSE	${\boldsymbol{\Delta }}{{\boldsymbol{H}}}_{{\bf{calc}}}^{{\boldsymbol{\theta }}}$	${\boldsymbol{\Delta }}{{\bf{G}}}_{{\bf{calc}}}^{{\boldsymbol{\theta }}}$	${{\boldsymbol{K}}}_{{\boldsymbol{e}}{\boldsymbol{q}}}^{{\bf{c}}{\bf{a}}{\bf{l}}{\bf{c}}}$
TFE–EO^c^	−30.0	5.3	0.9	−29.6	7.2	5.5 × 10^−2^
TFE–TMO	−35.2	5.4	0.9	−34.8	3.0	2.9 × 10^−1^
TFE–THF-1	−35.6	5.4	0.9	−35.0	2.6	3.6 × 10^−1^
TFE–THF-2	−35.4	5.5	1.0	−34.8	2.9	3.1 × 10^−1^
TFE–THP-1	−37.1	5.3	1.3	−36.8	2.6	3.6 × 10^−1^
TFE–THP-2	−36.0	4.9	1.2	−35.4	3.3	2.6 × 10^−1^

^a^All energies are in kJ mol^−1^. ^b^*BE* are corrected with ZPVE and BSSE. ^c^ref.^34^.

### OH-stretching transitions

**Figure 2:**
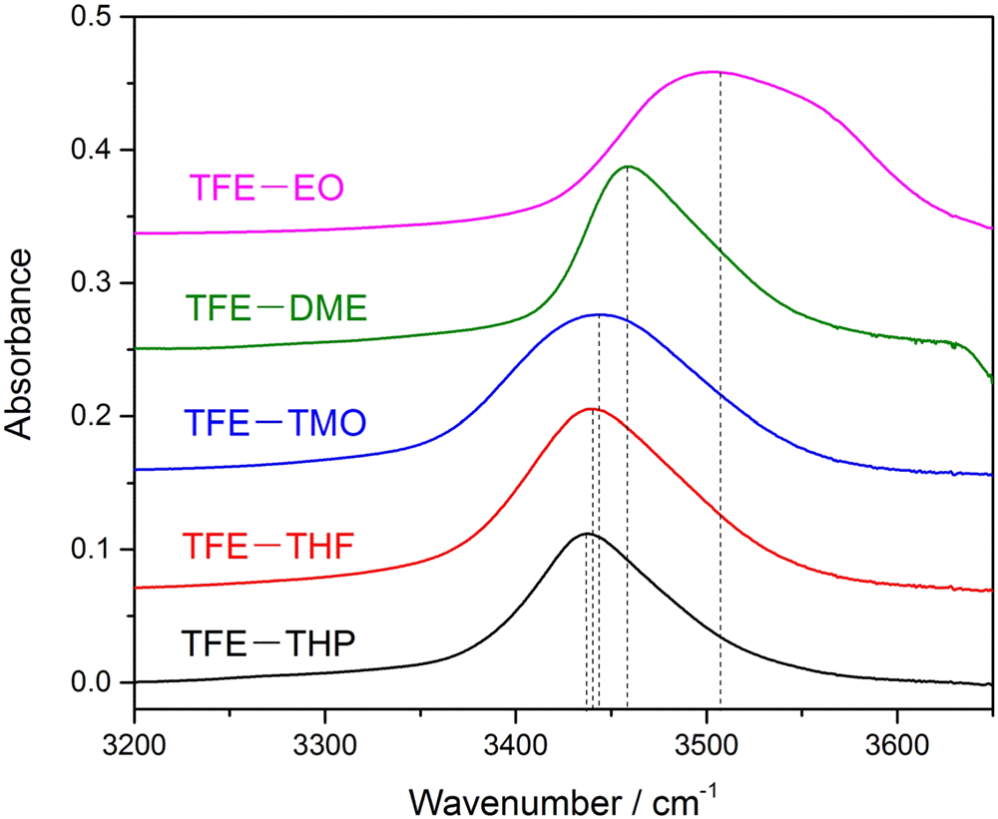
Spectra recorded with a 20 cm path length cell and different pressures: 10 Torr TFE + 77 Torr DME (green), 12 Torr TFE + 95 Torr EO (purple), 9 Torr TFE + 27 Torr TMO (blue), 12 Torr TFE + 28 Torr THF (red), 16 Torr TFE + 18 Torr THP (black). The spectra have been offset.

The formation of hydrogen bond complexes between TFE and cyclic ethers were probed by monitoring the evolution of the OH band in the FTIR spectra, shown in Fig. 2. A summary of the frequencies of complexes are listed in Table 3. The IR spectra of monomers and mixtures were recorded using a 20 cm long gas cell as shown in Figure S2. We obtained the spectra of the complexes in the OH-stretching transition region by subtracting two monomer spectra from that of mixture^30,35^. To check the reproducibility of the experiment, the spectra of individual components and that of the mixture spectra were recorded at different pressure combinations. It is notable that the strength of OH-stretching fundamental transitions increased with pressure, in agreement with observations form other studies^31,34^. The integrated absorbance of the OH-stretching band of the complexes, plotted against the product of the monomers pressures, shows a linear fit, indicating the formation of a 1:1 complex. (see Figure S3). The integration regions for TFE–TMO, TFE–THF and TFE–THP are 3197–3602, 3177–3626 and 3175–3626 cm^−1^, respectively.

**Table 3:** OH-stretching wavenumbers and oscillator strengths of the TFE and TFE− EO/TMO/THF/THP complexes, calculated with the B3LYP-D3/aug-cc-pVTZ method.

Conformer	$\tilde{{\boldsymbol{v}}}/{\bf{c}}{{\bf{m}}}^{-{\bf{1}}}$		${\boldsymbol{\Delta }}\tilde{{\boldsymbol{v}}}/{\bf{c}}{{\bf{m}}}^{{\boldsymbol{-}}1}$ ^**a**^		***f***	***f*** **/** ***f***_**TFE**_
	**Calculated**	**Observed**	**Calculated**	**Observed**		
TFE	3804	3657^b^			9.1 × 10^−6^	
TFE−EO^c^	3527	3502	277	155	1.5 × 10^−4^	16.7
TFE−TMO	3450	3444	353	213	2.0 × 10^−4^	21.6
TFE−THF-1	3453	3440	351	217	2.0 × 10^−4^	22.3
TFE−THF-2	3455	3440	349	217	2.0 × 10^−4^	22.1
TFE−THP-1	3461	3437	342	220	1.7 × 10−^4^	18.9
TFE−THP-2	3508	3437	296	220	1.6 × 10^−4^	17.1

^a^$${\rm{\Delta }}{\tilde{v}}_{{\rm{OH}}}={\tilde{v}}_{{\rm{TFE}}}-\tilde{v}{\rm{complex}}$$. ^b^ref.^35^. ^c^ref.^34^.

Similar to the TFE–EO complexes, the formation of TFE–TMO/THF/THP in the mixture before subtraction noticeable and, consequently, the complex bands were obtained by spectral subtraction. The OH-stretching vibrational bands of TFE–TMO, TFE–THF and TFE–THP were measured to be 3444, 3440 and 3437 cm^−1^, respectively. It can be seen that their OH-stretching fundamental transitions are close to each other, and the frequency increases as the ring size of the cyclic ether increases, with difference within 7 cm^−1^. Compared to the OH-stretching fundamental transitions of TFE–EO (3502 cm^−1^), the three-membered ring has larger frequency, indicating that the TFE–EO complex is less stable than others as mentioned before. In a previous study where the TFE–THF complex was detected in the gas phase, the mixing of TFE and THF caused a decrease in the intensity of the alcohol band centered at 3657 cm^−1^. The characteristic free OH stretching vibration, and the appearance of a new broader band around 3440 cm^−1^ due to a bonded OH-stretching vibration, are in good agreement with our experiment^11^.

The shift to lower frequencies of the OH-stretching bands relative to those in the corresponding monomers, reflecting the lengthening of these bonds as a result of the hydrogen bond formation, is a major indicator. In agreement with the elongation of the O-H bond discussed in the previous section, the OH-stretching band upon complexation appears shifted to lower frequency values. The OH-stretching vibrational wavenumbers and red shifts are shown in Table 3, where reported values of the gas-phase OH-stretching vibration bands of TFE are taken from our previous work^35^. The red shifting is relatively small for the three-membered ring systems (155 cm^−1^ for TFE–EO). In the other cases, the OH-stretching frequencies vary more significantly. In fact, for the four-, five-, six-membered rings, the variation is 213 cm^−1^ for TFE–TMO, 217 cm^−1^ for TFE–THF, and 220 cm^−1^ for TFE–THP. The correlation between the hydrogen bond basicity and infrared shifts has been determined for cyclic ethers, indicating that a general correlation does not exist for all kinds of bases. However, the basicity vs. red shift plot shows separate lines for six-, five-, four-, and three-membered rings ranging from right to left, which suggests the increased red shift with the increased ring size^24^. The red shifts are 103, 126, 150, 153 and 157 cm^−1^ for MeOH–cyclopropanone, MeOH–cyclobutanone, MeOH–cyclopentanone, MeOH–cyclohexanone and MeOH–cycloheptanone complexes, respectively^28^. Similarly, the enhancements of the CH-stretching band for complexations with the three ketones are estimated to be 5.0, 10.5, and 11.7 cm^−1^ for cyclohexanone, cyclopentanone, and cyclobutanone, respectively, and the order follows the sequence of blue shifting. Therefore, it can be anticipated that the electrostatic stabilization energy of the hydrogen bond complexes of chloroform with the said three cyclic ketones would follow the sequence cyclohexanone > cyclopentanone > cyclobutanone^26^. However, the red shift difference between TFE–TMO, TFE–THF and TFE–THP is very small (<7 cm^−1^). It is worth mentioning at the outset that one cannot expect complete agreement between the predictions of such gas phase calculations with the observed spectral changes for measurements performed at room temperature. With the size of ring increasing, the hydrogen bond complex is slightly more stable, but their stabilities are close. The deconvolution of the complexes absorption bands is shown in Figure S4, where the best fit of one Lorentzian function was obtained (see Supplementary information).

The OH-stretching vibrational bands of TFE and complexes are given in Tables 3 and S4, wherefrom it is seen that calculated wavenumbers and red shifts are larger than ones^34^. The calculated red shifts of the TFE–EO complex are smaller than those in other complexes, whereas the TFE–TMO complex has the largest red shift (353 cm^−1^), which is consistent with the differences in geometry parameters. The oscillator strengths (*f*) and relative intensities (*f*/*f*_TFE_) of the OH-stretching vibrational bands in the complexes are shown in Tables 3 and S4. The oscillator strengths of OH-stretching in the complexes were determined to be about 16–20 times stronger than that in TFE. The intensity increase is considered to be a criterion for hydrogen bonding^36^. As for the MeOH–dimethylamine and MeOH–trimethylamine, the OH-stretching fundamental transition intensities are calculated to be 51 and 61 times stronger than that of MeOH^31^. In the present study, the oscillator strengths of complexes do not change significantly with increasing ring size of the cyclic ether. Likewise, these oscillator strengths are quite similar for conformers of a given complex.

### Thermodynamic equilibrium constant

The equilibrium constant (*K*_p_) for the complex formation was determined as:

1 $${K}_{p}=\frac{{p}_{{\rm{complex}}}/{p}^{\theta }}{{p}_{{\rm{TFE}}}/{p}^{\theta }\times {p}_{\mathrm{TMO}/\mathrm{THF}/\mathrm{THP}}/{p}^{\theta }}$$

where *p*^θ^ is the standard pressure (1 bar = 0.99 atm). *p*_i_’s are the pressures of the complex, TFE and cyclic ethers, measured before measuring the pressure of the mixture. The partial pressure (Torr) of the complex was determined from the measured integrated absorbance and calculated oscillator strengths (*f*_calc_) of the fundamental OH-stretching band, as follows^37^:

2 $${p}_{{\rm{complex}}}=2.6935\times {10}^{-9}({{\rm{K}}}^{-{\rm{1}}}\,{\rm{Torr}}\,{\rm{m}}\,{\rm{cm}})\frac{T\int A(\tilde{v})\,d\tilde{v}}{f{\rm{calc}}\,\times \,l\,}$$

where *p*_complex_ is in Torr, *T* is the absolute temperature in K, ${\int }^{}A(\tilde{v})d\tilde{v}$ is the integrated absorbance in cm^−1^, *f*_calc_ is the calculated intensity and *l* is the optical path length in m. The numerical values for *f*_calc_ are given in Table 3. This method to determine the complex partial pressure has been widely used in similar previous studies^30,31,38,39^.

**Figure 3:**
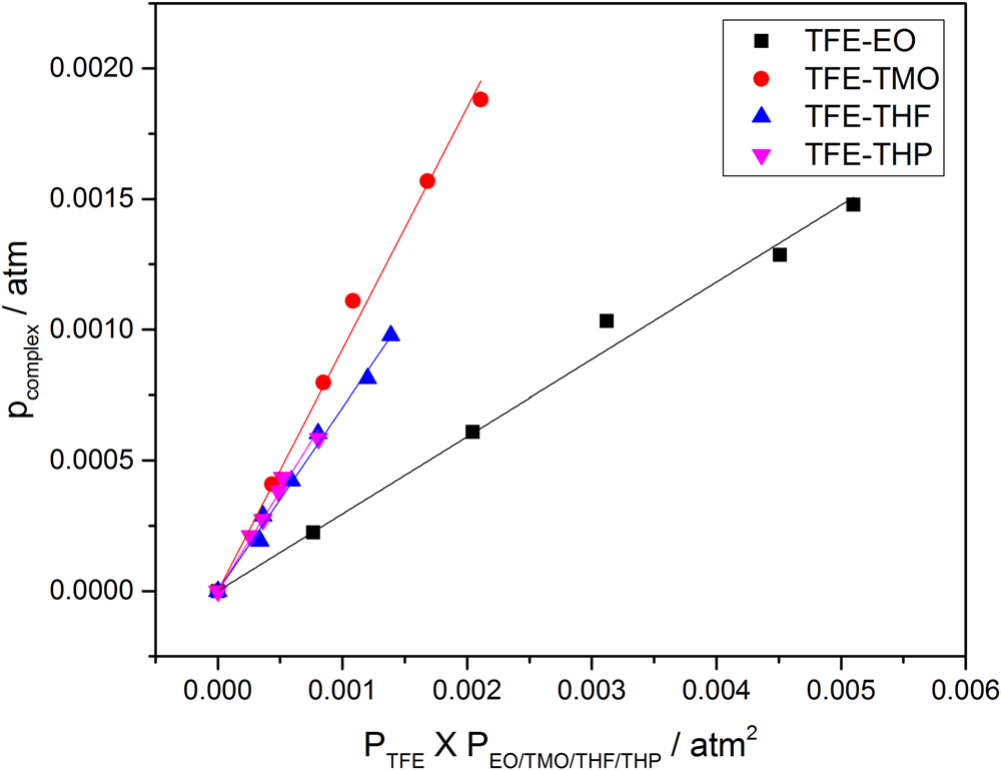
Plot of *p*_complex_ against *p*_TFE_ × *p*_EO/TMO/THF/THP_.

Considering the TFE–THF/THP complexes, two of the conformers of each complex type have very similar binding energies. Therefore, the average of the calculated oscillator strengths of the two conformers of each complex type was used to determine the pressures of complexes. The *f*_calc_ used were 2.0 × 10^−4^, 2.0 × 10^−4^ and 1.65 × 10^−4^ for TFE–TMO, TFE–THF and TFE–THP, respectively. The TFE–TMO/THF/THP complex pressure is plotted against *p*_TFE_ × *p*_TMO/THF/THP_ in Fig. 3, wherefrom *K*_p_ was obtained from the slope of the least-square fitting of the data. The measured *K*_p_ for TFE–TMO, TFE–THF and TFE–THP are 9.3 × 10^−1^, 7.0 × 10^−1^ and 7.7 × 10^−1^, respectively, which are all larger than that of the TFE–EO complex. If we assume that the intensity of the OH-stretching fundamental transition is overestimated by a factor of two, similar to the methanol–dimethylamine complex, we get *K*_p_ values of 1.86, 1.40 and 1.54 for TFE–TMO, TFE–THF and TFE–THP, respectively^31^. *K*_p_ represents the ability of formation of complex, in this case, these *K*_p_ values show, not surprisingly, that three complexes have basically equivalent stabilities and hydrogen bond strengths. Compared with the ${K}_{eq}^{{\rm{calc}}}$ obtained from theoretical calculations, the B3LYP-D3/aug-cc-pVTZ method predicts most stable complexes (see Tables 2 and S3). In general, other calculated values are significantly underestimated by theoretical calculations. The Gibbs free energies of formation, ${\rm{\Delta }}{G}_{\text{expt}}^{\theta }$, determined from the measured *K*_p_ values were found to be 0.2, 0.9 and 0.7 kJ mol^−1^ for TFE–TMO, TFE–THF and TFE–THP, respectively. Compared to the computed Gibbs free energies, the B3LYP-D3/aug-cc-pVTZ results predict the closest values to the experimental values.

### AIM analysis

AIM analysis has been carried out to gain further insights into the nature of the weak interactions of these clusters. Figure S5 demonstrates distinctly the bond critical point (BCP), ring critical point (RCP) and the bond path corresponding to preferable interactions. According to the topological analysis of electron density in the theory of AIM, there are three fundamental criteria to judge the existence of a hydrogen bond: (i) the existence of BCP; (ii) the electron density *ρ*(r) ranges from 0.002–0.040 a.u.; and (iii) the Laplacian ∇^2^*ρ*(r) value falls in the range of 0.024–0.139 a.u. From the data listed in Tables 4 and S5, positive values of ∇^2^*ρ*(r) and reasonably small *ρ*(r) values verify the existence of hydrogen bonding interactions in TFE–cyclic ethers complexes. The *ρ*(r) in this study ranges from 0.0393 to 0.0426 a.u. and the values exceed (<0.003 a.u.) the upper value of the criteria, which indicates stronger hydrogen bonds formed^40^. Previous studies revealed that for the organic acid–sulfuric acid system, topological analysis employing AIM shows that the charge density and the Laplacian at BCP of the hydrogen bonds of the benzoic acid–sulfuric acid and cis-pinonic acid–sulfuric acid are 0.07 and 0.16 a.u., respectively, which falls in or exceeds the range of one strong and one medium-strength hydrogen bonding criteria^41^. Specifically, in the TFE–TMO/THF/THP complexes, the ∇^2^*ρ*(r) values fall within the proposed range, evidencing the existences of O–H···O hydrogen bonds. Besides, the slightly positive values of ∇^2^*ρ*(r) suggest moderately strong hydrogen bonding, which provides a stable structure of TFE–TMO/THF/THP. The *ρ*(r) (<0.006 a.u.) and ∇^2^*ρ*(r) (<0.005 a.u.) differences for all complexes are not significant (shown in Table 4), indicating that there is no big variation with the different ring size and this phenomenon is consistent with the geometric and experimental results.

**Table 4:** Change in electronic charge at H atom Δ*q*(H), change in atomic energy at H atom Δ*E*(H), electron density *ρ*(r) and Laplacian ∇^2^*ρ*(r) at the BCPs for the complexes. Calculations were performed at the B3LYP-D3/aug-cc-pVTZ level of theory. All values are in a.u.

Conformer	Δ*q*(H)	Δ*E*(H)	*ρ*(BCP)	∇^2^*ρ*(BCP)
TFE−EO^a^	0.0386	0.0242	0.0369	0.0954
TFE−TMO	0.0392	0.0244	0.0424	0.0979
TFE−THF-1	0.0401	0.0248	0.0426	0.0988
TFE−THF-2	0.0405	0.0251	0.0425	0.0985
TFE−THP-1	0.0369	0.0236	0.0405	0.0945
TFE−THP-2	0.0410	0.0251	0.0393	0.0995

^a^ref.^34^.

The change in atomic energy (Δ*E*(H)) and the change in electronic charge (Δ*q*(H)) at the H atom constitute an example of synergy between them^28^. For the H atom, this energy increases upon complexation (Δ*E*(H) > 0)^42^. A charge transfer from the proton acceptor to the proton donor should be expected for hydrogen bond complex. It can be observed from the data of Table 4 that the charge transferred to TFE is relatively smaller for the smallest ring. It is worth mentioning that, as expected, unconventional C–H···F hydrogen bonds exhibit rather large bond distances, typically greater than 2.5 Å. In respect to the TFE–TMO/THF/THP, unconventional hydrogen bonds were found between the fluorine of TFE and the hydrogen in the methyl group of cyclic ethers. All these hydrogen bonds are characterized by the existence of a BCP between the hydrogen of the hydrogen acceptor and the heteroatom of the hydrogen bond donor.

### NBO analysis

**Figure 4:**
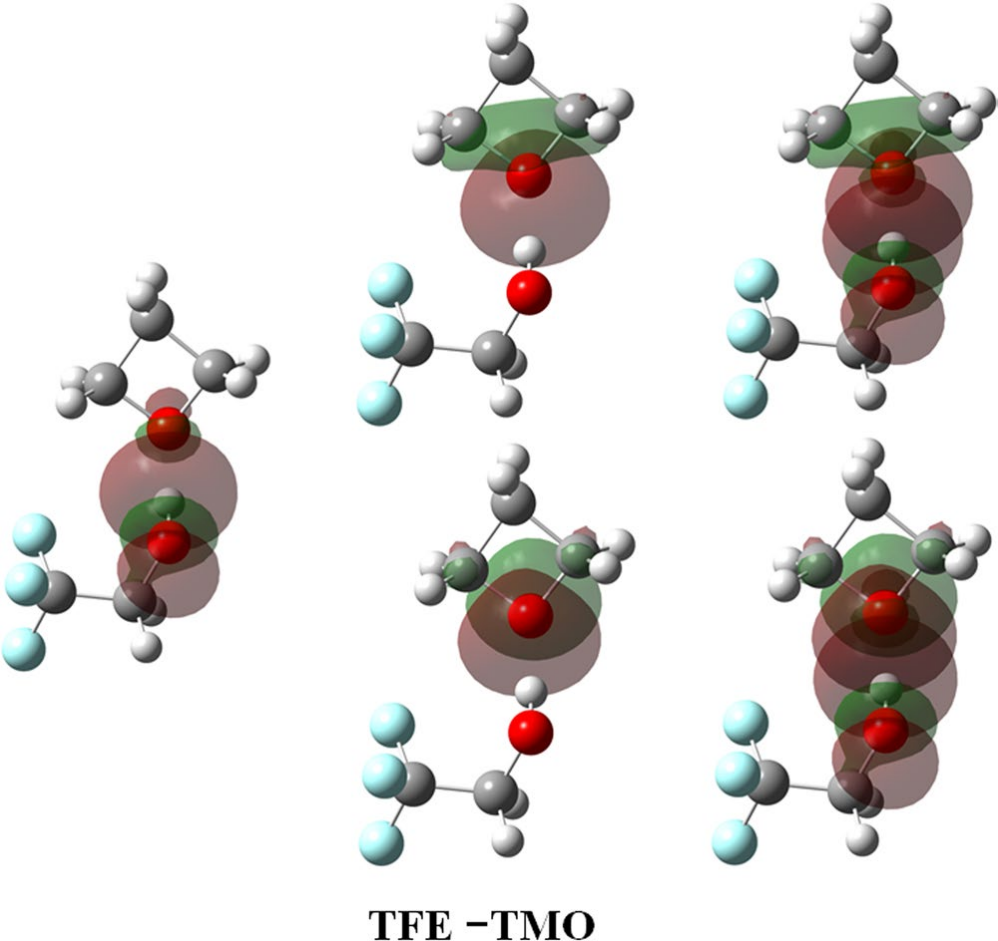
The hydrogen bond donor NBO (on the left), acceptor NBO (in the middle), and interacting donor-acceptor NBOs (*n*_pO_ → σ*_O-H_, on the right) of the TFE−TMO. For the complex, both *n*_sp2O_ → σ*_O-H_ (top) and *n*_pO_ → σ*_O-H_ (bottom) are shown.

NBO analysis is beneficial for understanding molecular cluster formation from the local orbital interactions between the donor and the acceptor. In particular, the second order perturbative energy (${E}_{i\to j\ast }^{(2)}$) provides a measure of the overlap integral between the lone-pair orbital of the acceptor and the antibonding orbital of the donor (where i and j^*^ stand for a lone pair orbital and an antibonding σ^*^ or π^*^ orbital, respectively). This is helpful to estimate the energy loss caused by electron delocalization in weak intermolecular interactions, and hence beneficial for evaluating the contribution of a certain interaction to the cluster stability. The typical results of NBO analyses for TFE–cyclic ethers complexes are presented in Tables 5 and S6, including the changes in the natural charges on H (Δ*q*(H)) and O (Δ*q*(O)) atoms, the occupancy in the p-type lone pair orbital (*δ*(*n*_pO_)), the occupancy in the antibonding orbital (*δ*(σ*_O-H_)), the second-order perturbation energy (${E}_{i\to j\ast }^{(2)}$), the zeroth-order energy difference between the lone pair orbital and antibonding orbital (${\varepsilon }_{j\ast }^{(0)}-{\varepsilon }_{i}^{(0)}$), and the Kohn-Sham matrix element between the orbitals ($\langle {\phi }_{i}^{(0)}|{\hat{F}}_{KS}|{\phi }_{j\ast }^{(0)}\rangle$), from which one can clearly see the interaction orbitals and their interaction strength. Figure 4 presents the typical NBO pattern of TFE–TMO complex, that is, charge transfer delocalization interactions of the lone pair orbitals on the oxygen atoms over the antibonding orbitals of the O–H bonds in the TFE monomer, and the typical NBO orbital pattern of other complexes are in the electronic supplementary information. It shows that the two lone pairs of the oxygen atom contribute to the hydrogen bond, *n*_sp2O_ → σ*_O-H_ and *n*_pO_ → σ*_O-H_. Their contributions are, however, not equal because of their different orientations with respect to σ^*^_O-H_.

**Table 5:** NBO parameters for the TFE complexes, calculated with the B3LYP-D3/aug-cc-pVTZ method^a^.

NBO parameters	TFE−EO	TFE−TMO	TFE−THF-1	TFE−THF-2	TFE−THP-1	TFE−THP-2
Δ*q*(H)	0.01820	0.02100	0.02243	0.02244	0.01753	0.02602
Δ*q*(O)	−0.03114	−0.02656	−0.02752	−0.02639	−0.03223	−0.03829
*δ*(*n*_*p*O_)	1.983,1.916	1.972,1.917	1.956,1.920	1.956,1.919	1.957,1.912	1.948,1.924
*δ*(σ*_O−H_)	0.0344	0.0406	0.0398	0.0396	0.0414	0.0351
${E}_{i\to {j}^{\ast }}^{(2)}$	55.31	70.29	69.54	68.91	61.33	57.83
	(11.59 + 43.72)	(19.54 + 50.75)	(15.36 + 54.18)	(15.23 + 53.68)	(13.97 + 47.36)	(32.01 + 25.82)
${\varepsilon }_{{j}^{\ast }}^{(0)}-{\varepsilon }_{i}^{(0)}$	1.97 (1.19 + 0.78)	1.86 (1.07 + 0.79)	1.80 (0.98 + 0.82)	1.81 (0.99 + 0.82)	1.79 (0.99 + 0.80)	1.81 (1.00 + 0.81)
$\langle {\phi }_{i}^{(0)}|{\hat{F}}_{KS}|{\phi }_{{j}^{\ast }}^{(0)}\rangle$	0.133 (0.051 + 0.082)	0.151 (0.063 + 0.088)	0.147 (0.054 + 0.093)	0.146 (0.054 + 0.092)	0.137 (0.051 + 0.086)	0.142 (0.078 + 0.064)

^a^The values in the parentheses give the individual contribution of the nonbonding orbitals of oxygen. The *δ*(*n*_pO_) values are for each of the two lone pairs. ${E}_{i\to {j}^{\ast }}^{(2)}$ is in kJ mol^−1^, all other values are in a.u.

The ${E}_{i\to j\ast }^{(2)}$ values for the TFE–TMO/THF-1/THF-2/THP-1/THP-2 complexes are 70.29, 69.54, 68.91, 61.33 and 57.83 kJ mol^−1^, respectively. A relatively large ${E}_{i\to j\ast }^{(2)}$ value suggests strong charge transfer interaction responsible for the traditional hydrogen bond. This is mainly ascribed to two aspects. On the one hand, the electrostatic attraction between the higher electronegativity Y and the positive H elongates the X–H bond^43^. In the case of TFE–TMO, the charges on the atoms H are all 0.471e, but charges on O are −0.575e, exhibiting a strong electrostatic attraction interaction. On the other hand, the charge transfer or hyperconjugative interactions between the proton donor and the proton acceptor enlarge the X–H bond length, and hence weaken interactions^44,45^. For the TFE–TMO complex, the changes of natural atom charges on the proton H and proton acceptor O are 0.02100e and −0.02656e, respectively, suggesting that the cluster is stabilized by the classical red shifted hydrogen bonding. In addition, for the isolated TFE monomer, the σ^*^_O-H_ population is 0.0069e. With regard to TFE–TMO complex, the σ^*^_O-H_ population is 0.0406e; the increase in antibonding is consistent with the stretching characteristic of O–H bonds, which implies a redshift of the stretching vibrational mode. In regard to the NBO orbitals of other complexes, it is found that TFE–THF/THP have similar increase in antibonding that proved the formation of complexes, and the TFE–TMO/THF/THP complexes are slightly more strongly bound than TFE–EO (${E}_{i\to {j}^{\ast }}^{(2)}$ = 55.31 kJ mol^−1^). However, the ${E}_{i\to {j}^{\ast }}^{(2)}$ differences for the complexes containing different hydrogen bond acceptors are small and can be neglected.

## Conclusions

In summary, we have studied the detailed structures and stability of the TFE–cyclic ethers complexes to explore the ring-size effects on the hydrogen bonded complexes, by using gas phase FTIR and density functional theory calculations. The stable structures for all complexes are obtained. The observed red shifts of the OH-stretching transition provide information about the change of the bands upon complexation and can be used as a fingerprint to identify these complexes. The shifts increase with increasing ring size, but the change is not substantial. For the TFE–TMO, TFE–THF and TFE–THP complexes, these red shifts were observed at 213, 217 and 220 cm^−1^, respectively. The electron densities and Laplacian of the electron densities of the O–H···O in the TFE–EO complex being smaller than in other complexes indicate weaker hydrogen bonds in small rings than in bigger ones. Considering all the effects on the hydrogen bond, it is concluded that the hydrogen bonds are of similar strength with the ring size, based on the energy calculations and the AIM, and NBO analyses.

### Experimental details

Prior to their use, TFE (Aladdin, anhydrous, 99.5%), TMO (Alfa, 97%), THF (Adamas, 99.5%) and THP (Adamas, 98%+) were purified with several freeze-pump-thaw cycles on vacuum condition. The infrared spectra were obtained using a FTIR spectrometer (Bruker Vertex 70), employing a DLaTGS (deuterated and lanthanum α alanine doped triglycine sulfate) detector and a KBr beam splitter, co-adding 128 scans at 1.0 cm^−1^ resolution. The 20 cm long glass cell used for measuring the spectra of gases consisted of a pair of CaF_2_ windows and measurements have been performed at room temperature. The gas pressures were measured using Tamagawa CDG-800 pressure gauges, which kept the base pressure lower than 1 × 10^−4^ Torr. We waited approximately 30 min for the sample in the cell to reach a stable temperature prior to each measurement. The OPUS program was used throughout all of the measurements to perform spectral subtraction and band integration.

## Methods

### Computational details

To confirm the observed bands in the vibrational spectra and to obtain further insights into the hydrogen bond behavior, quantum chemical calculations are essential and were carried out in the present study using different approaches^30,31,39,46^. Uncertainties are intrinsic to the method used and are entirely derived from the estimated uncertainties on the physical input parameters^47^. The performance of density functional theory (DFT) in reproducing molecular equilibrium geometries and the thermochemistry of atmospherically relevant prenucleation clusters has been investigated and it was demonstrated that all DFT results should be handled with care when modeling nucleation^48^. For an overview of the hydrogen bonded dimers, DFT were performed, but geometry optimization shows that dispersion interactions play an essential role in the aggregation process^49^. To carry these effects over into vibrational spectra, the B3LYP-D3 functional, which includes an empirical dispersion correction to noncovalent interaction^50^, was used in conjunction with the aug-cc-pVTZ basis set. The B3LYP functional was also used for comparison. To obtain reliable vibrational frequencies and thermochemistry for the complexes, the “opt = verytight” and “integral = ultrafine” convergence criteria were used during geometry optimization of the monomers and complexes. All molecular structures were confirmed as local minima by the absence of imaginary vibrational frequencies. All geometry optimizations and vibrational frequency calculations were performed using the Gaussian 09 program^51^.

Zero-point vibrational energies (ZPVE) and the basis set superposition errors (BSSE) were calculated to correct the electronic energies. The counterpoise (CP) procedure was used to remove BSSE^52^, which resulted in an improvement of the binding energies of weakly bound complexes^53–56^. It has been found that CP corrections are important when the geometry of binary complexes is optimized with small basis sets. If larger basis sets are used, this effect is minor and the error reduces to <1 kcal mol^−1^ for the binding energy^57,58^. It was noted that the interaction energies of the TFE–TMO/THF/THP complexes obtained at the B3LYP level of theory differ from those calculated when using the functional with dispersion correction. In view of this, all the following discussions are mainly based on the B3LYP-D3/aug-cc-pVTZ results.

Topological properties of the electronic density were characterized by using the AIM theory, which is well known to provide a thorough understanding of various molecular interactions, including hydrogen bond interactions^59,60^. The AIM analysis was performed using the AIM2000 program (version 2) package^61,62^. The NBO analysis was carried out to explain the hydrogen bonding as the donor-acceptor charge delocalization takes place between the lone pair of the acceptor and antibonding orbital of the donor^44,63^. The NBO analysis was performed with the B3LYP and B3LYP-D3 functionals for comparison.

### Data Availability

Datasets generated during and/or analysed during the current study are available from the corresponding author on reasonable request.

## Electronic supplementary material

Supplementary information
