# Supplementary material for: Ring-Size Effects on the Stability and Spectral Shifts of Hydrogen Bonded Cyclic Ethers Complexes
Source: Sci Rep. 2018 Jan 24;8:1553. doi: 10.1038/s41598-017-18191-3 (PMC5784011; doi:10.1038/s41598-017-18191-3)
Supplement: Supplementary file 1 — Supplementary information [file 41598_2017_18191_MOESM1_ESM.pdf]

Supplementary information

Ring-Size Effects on the Stability and Spectral Shifts of Hydrogen Bonded Cyclic Ethers Complexes

Shanshan Tang, Narcisse T. Tsona, Lin Du*

Environment Research Institute, Shandong University, Shanda South Road 27, 250100 Shandong, China

e-mail: lindu@sdu.edu.cn

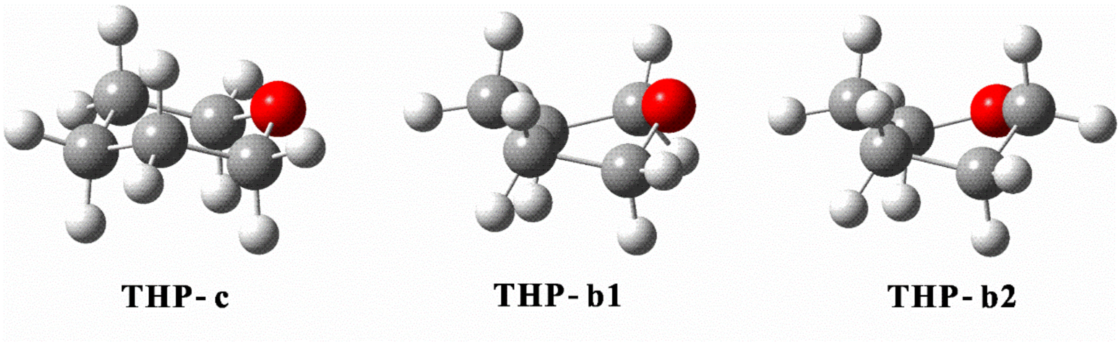

Figure S1. Structures of the THP monomers optimized using the B3LYP-D3/aug-cc-pVTZ method.

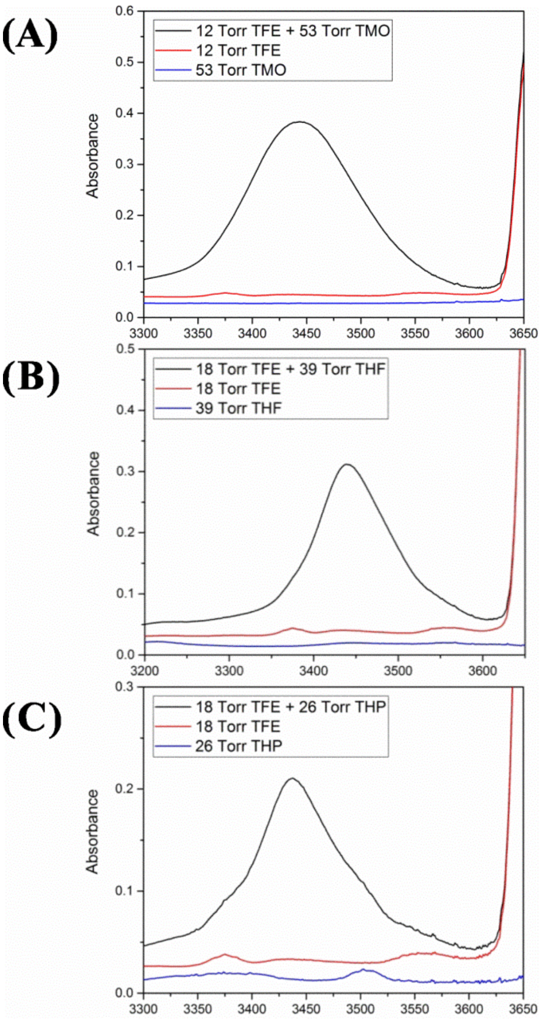

Figure S2. (A) Spectra of TFE, TMO, and their mixture in the 3300-3650 cm^{-1} region. (B) Spectra of TFE, THF, and their mixture in the 3200-3650 cm^{-1} region. (C) Spectra of TFE, THP, and their mixture in the 3300-3650 cm^{-1} region. A 20 cm path length cell was used.

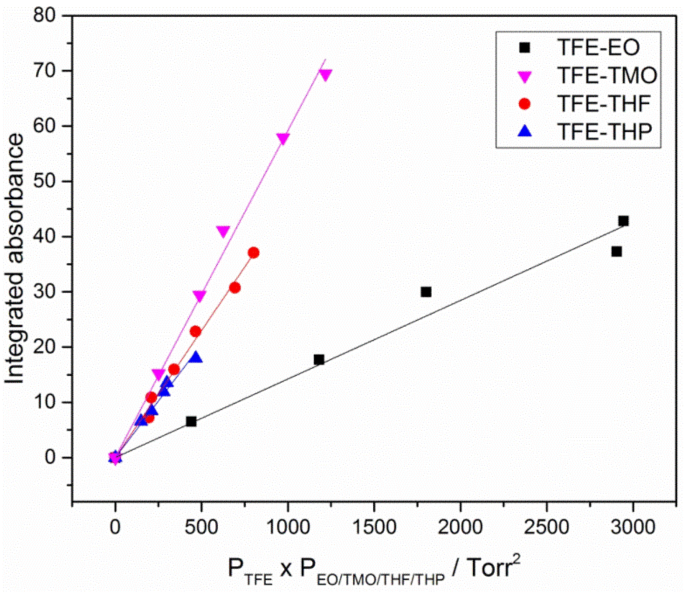

Figure S3. The integrated absorbance of the OH-stretching band in the TFE−EO, TFE−TMO, TFE−THF and TFE−THP complexes as a function of the product of the TFE and EO/TMO/THF/THP pressures. A 20 cm path length cell was used. The integration regions for TFE−EO, TFE−TMO, TFE−THF and TFE−THP are 3219-3659, 3197-3602, 3177-3626 and 3175-3626 cm^{-1}, respectively.

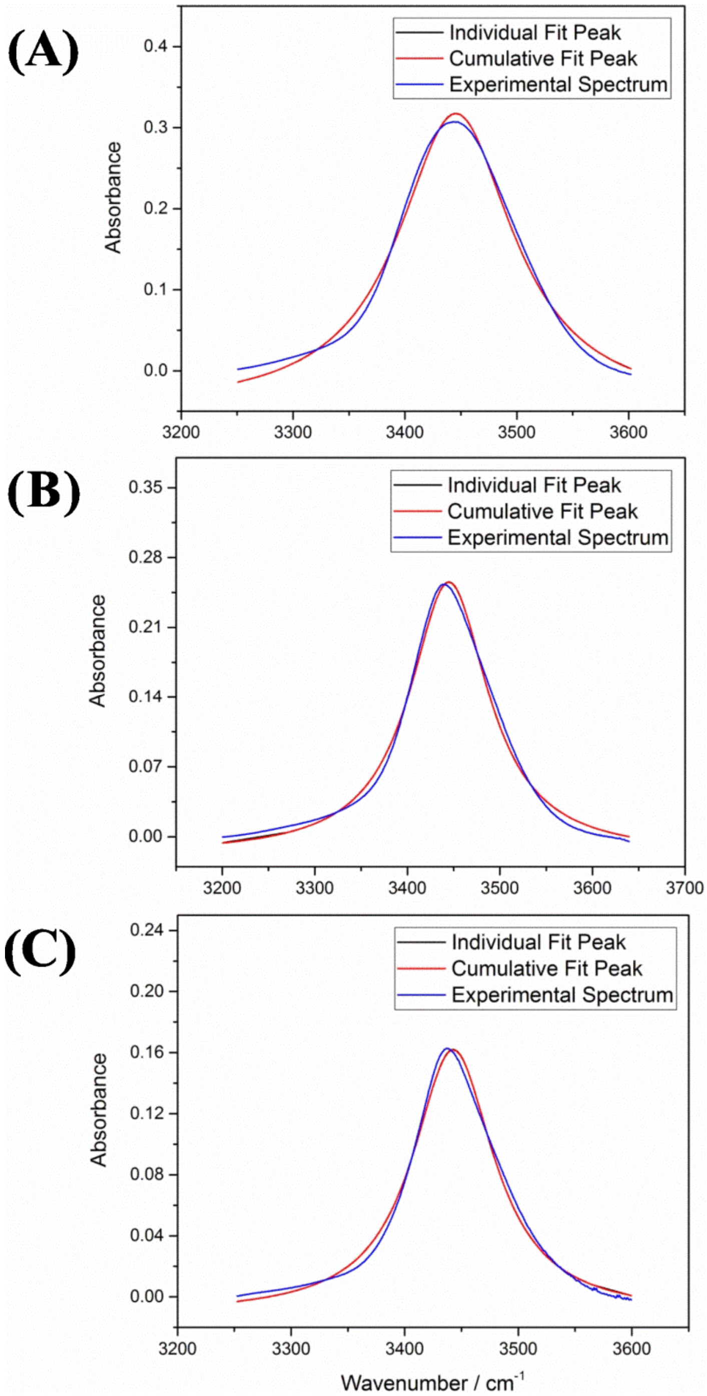

Figure S4. The deconvolution fittings of the OH-stretching fundamental transition bands of TFE−TMO (A), TFE−THF (B) and TFE−THP (C). The experimental and fitted bands for the complexes are denoted by blue and red curves, respectively.

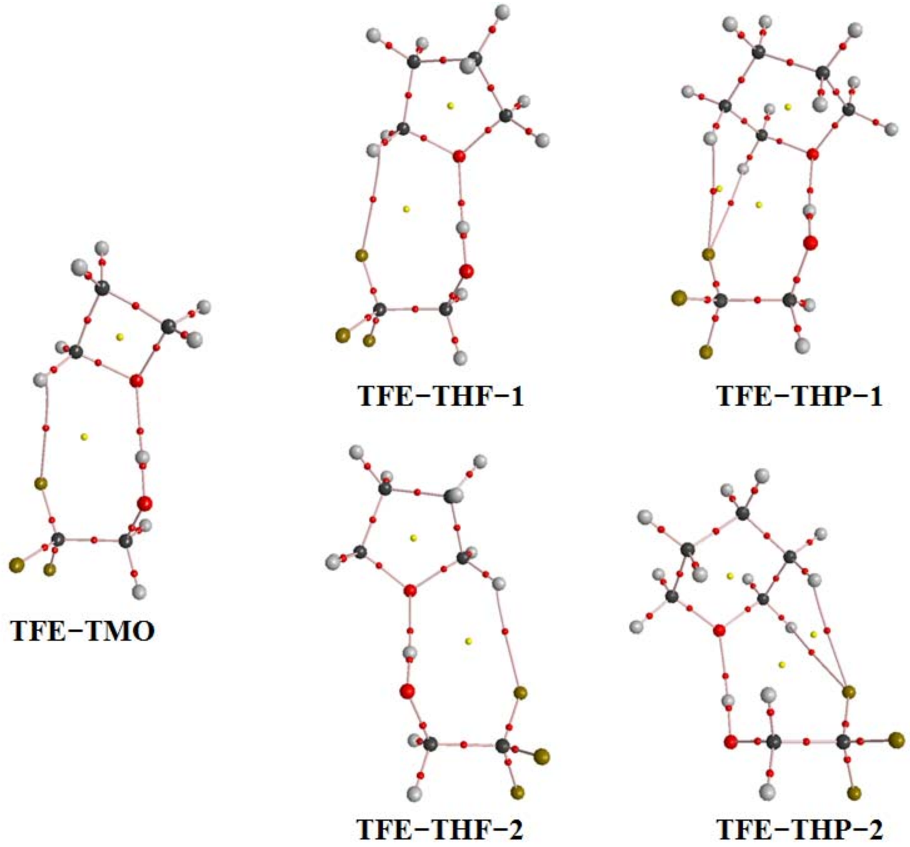

Figure S5. AIM plots of the complexes obtained with the B3LYP-D3/aug-cc-pVTZ method. The bond critical points and ring critical points are presented by the red and yellow balls, respectively.

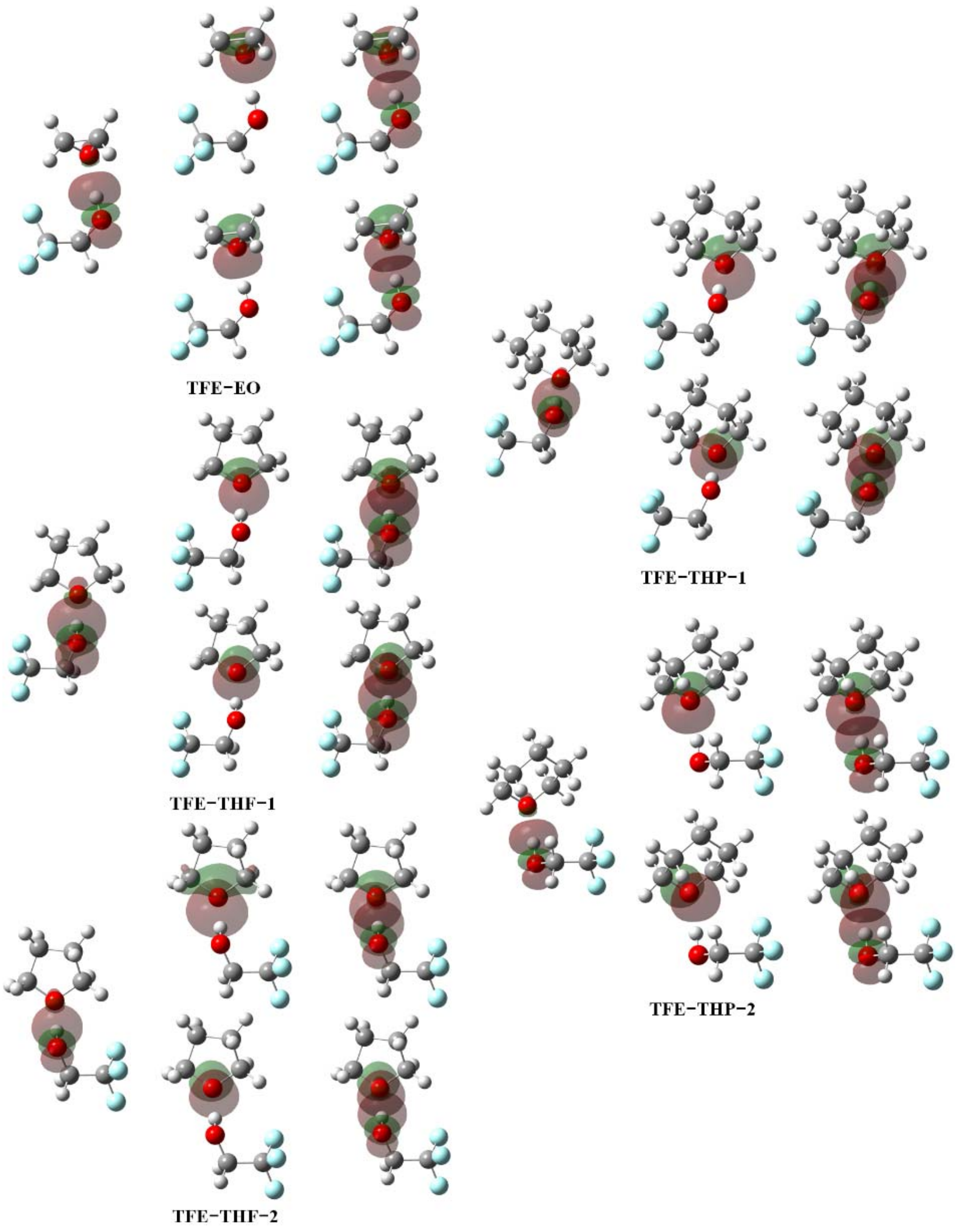

Figure S6. The hydrogen bond donor NBO (on the left), acceptor NBO (in the middle), and interacting donor-acceptor NBOs ($n_{pO}\rightarrow\sigma^*_{O\text{-}H}$, on the right) of the TFE−EO, TFE−THF and TFE−THP. For all the complexes, both $n_{sp2O}\rightarrow\sigma^*_{O\text{-}H}$ (top) and $n_{pO}\rightarrow\sigma^*_{O\text{-}H}$ (bottom) are shown.

Table S1. Calculated relative energy (relative to the energy of THP-c, *RE* in kJ mol^{-1}) of the THP isomers with the B3LYP and B3LYP-D3 functionals and the aug-cc-pVTZ basis set.

Isomer	Methods	*RE*
THP-c	B3LYP	0.0
	B3LYP-D3	0.0
THP-b1	B3LYP	24.2
	B3LYP-D3	24.8
THP-b2	B3LYP	27.0
	B3LYP-D3	26.8

Table S2. Optimized geometric parameters of the TFE−cyclic ether complexes, calculated at the B3LYP/aug-cc-pVTZ level of theory. Angles are in degrees and bond lengths are in Å.

Conformer	$r_{(OH)}$[a]	$\Delta r_{(OH)}$[b]	$r_{(HB)}$[c]	$\theta_{(HB)}$[d]
TFE−EO [e]	0.9750	0.0121	1.8294	172.9
TFE−TMO	0.9780	0.0151	1.7835	176.9
TFE−THF-1	0.9778	0.0149	1.7830	175.3
TFE−THF-2	0.9777	0.0149	1.7840	175.3
TFE−THP-1	0.9767	0.0138	1.7975	174.4
TFE−THP-2	0.9767	0.0138	1.7973	174.9

[a] OH bond length. [b] $\Delta r_{(OH)} = r_{complex} - r_{TFE}$, is the change in the OH bond length upon complexation. [c] Intermolecular hydrogen bond distance. [d] Intermolecular hydrogen bond angle, i.e., $\theta_{(O\text{–}H\cdots O)}$. [e] Ref [1]

Table S3. Binding energy (BE), enthalpy of formation ($\Delta H^{\theta}_{\text{calc}}$ at 298 K), Gibbs free energy of formation ($\Delta G^{\theta}_{\text{calc}}$ at 298 K) and equilibrium constant (K^{calc}_{eq} at 298 K) for the TFE−EO/TMO/THF/THP complexes. Calculations were performed with the B3LYP/aug-cc-pVTZ method [a]

Conformer	*BE* [b]	ZPVE	BSSE	$\Delta H^{\theta}_{\text{calc}}$	$\Delta G^{\theta}_{\text{calc}}$	$K^{\text{calc}}_{\text{eq}}$
TFE−EO [c]	-21.1	4.7	0.7	-20.2	14.7	2.7×10^{-3}
TFE−TMO	-25.6	4.8	0.8	-24.6	10.3	1.6×10^{-2}
TFE−THF-1	-25.1	4.7	0.8	-23.9	10.0	1.7×10^{-2}
TFE−THF-2	-25.0	4.8	0.8	-23.9	11.2	1.1×10^{-2}
TFE−THP-1	-24.2	4.5	0.9	-22.9	12.2	7.3×10^{-3}
TFE−THP-2	-24.0	4.4	0.9	-22.7	11.7	8.8×10^{-3}

[a] All energies are in kJ mol^{-1}. [b] *BE* are corrected with ZPVE and BSSE. [c] Ref [1].

Table S4. OH-stretching wavenumbers (in cm^{-1}) and oscillator strengths of the TFE and TFE−EO/TMO/THF/THP complexes, calculated with the B3LYP/aug-cc-pVTZ method

Conformer		B3LYP
TFE	$\tilde{\nu}$	3806
	f_{TFE}	9.2×10^{-6}
TFE−EO [b]	$\tilde{\nu}$	3553
	$\Delta\tilde{\nu}^{b}$	253
	f	1.5×10^{-4}
	f/f_{TFE}	16.5
TFE−TMO	$\tilde{\nu}$	3492
	$\Delta\tilde{\nu}^{b}$	314
	f	1.9×10^{-4}
	f/f_{TFE}	20.7
TFE−THF-1	$\tilde{\nu}$	3496
	$\Delta\tilde{\nu}^{b}$	310
	f	1.9×10^{-4}
	f/f_{TFE}	21.3
TFE−THF-2	$\tilde{\nu}$	3497
	$\Delta\tilde{\nu}^{b}$	309
	f	1.9×10^{-4}
	f/f_{TFE}	21.2
TFE−THP-1	$\tilde{\nu}$	3518
	$\Delta\tilde{\nu}^{b}$	288
	f	1.8×10^{-4}
	f/f_{TFE}	19.7
TFE−THP-2	$\tilde{\nu}$	3519
	$\Delta\tilde{\nu}^{b}$	287
	f	1.9×10^{-4}
	f/f_{TFE}	21.0

[a] $\Delta\tilde{\nu}_{OH} = \tilde{\nu}_{TFE} - \tilde{\nu}_{complex}$. [b] Ref [1].

Table S5. AIM parameters for the complexes with the B3LYP/aug-cc-pVTZ method (all values in a.u.)

Conformer	Δq(H)	ΔE(H)	ρ(BCP)	$\nabla^2\rho$(BCP)
TFE−EO [a]	0.0375	0.0240	0.0340	0.0908
TFE−TMO	0.0424	0.0275	0.0382	0.0950
TFE−THF-1	0.0408	0.0264	0.0381	0.0957
TFE−THF-2	0.0420	0.0272	0.0381	0.0955
TFE−THP-1	0.0424	0.0280	0.0365	0.0947
TFE−THP-2	0.0391	0.0252	0.0366	0.0945

[a] Ref [1].

Table S6. NBO parameters for the TFE complexes, calculated at the B3LYP/aug-cc-pVTZ level of theory [a]

NBO parameters	TFE−EO	TFE−TMO	TFE−THF-1	TFE−THF-2	TFE−THP-1	TFE−THP-2
Δq(H)	0.02087	0.02375	0.02530	0.02535	0.02577	0.02593
Δq(O)	-0.03562	-0.02902	-0.03006	-0.02989	-0.03821	-0.03762
$\delta(n_{pO})$	1.984,1.919	1.970,1.923	1.953,1.926	1.953,1.926	1.950,1.924	1.952,1.921
$\delta(\sigma^*_{O\text{–}H})$	0.0310	0.0358	0.0349	0.0348	0.0333	0.0330
$E^{(2)}_{i\rightarrow j^*}$	48.87 (12.80+36.07)	58.99 (23.47+35.52)	58.53 (20.25+38.28)	58.33 (20.00+38.33)	52.00 (27.57+24.43)	53.51 (24.77+28.74)
$\varepsilon^{(0)}_{j^*} - \varepsilon^{(0)}_{i}$	1.98 (1.19+0.79)	1.87 (1.08+0.79)	1.81 (0.99+0.82)	1.81 (0.99+0.82)	1.81 (1.01+0.80)	1.81 (1.01+0.80)
$\langle \varphi^{(0)}_i \mid \hat{F}_{KS} \mid \varphi^{(0)}_{j^*} \rangle$	0.129 (0.054+0.075)	0.144 (0.070+0.074)	0.140 (0.062+0.078)	0.140 (0.062+0.078)	0.135 (0.073+0.062)	0.136 (0.069+0.067)

[a] The values in the parentheses give the individual contribution of the nonbonding orbitals of oxygen. The $\delta(n_{pO})$ values are for each of the two lone pairs. $E^{(2)}_{i\rightarrow j^*}$ is in kJ mol^{-1}, all other values are in a.u.

References

1 Tang, S., Zhao, H. & Du, L. Hydrogen Bonding in Alcohol-ethylene Oxide and Alcohol-ethylene Sulfide Complexes. *RSC Adv.* **6**, 91233-91242, doi:10.1039/c6ra16205c (2016).
